# Supplementary material for: Dithiothreitol Affects the Fertilization Response in Immature and Maturing Starfish Oocytes
Source: Biomolecules. 2023 Nov 17;13(11):1659. doi: 10.3390/biom13111659 (PMC10669828; doi:10.3390/biom13111659)
Supplement: Supplementary file 1 [file biomolecules-13-01659-s001.zip › biomolecules-2657502-supplementary.pdf]

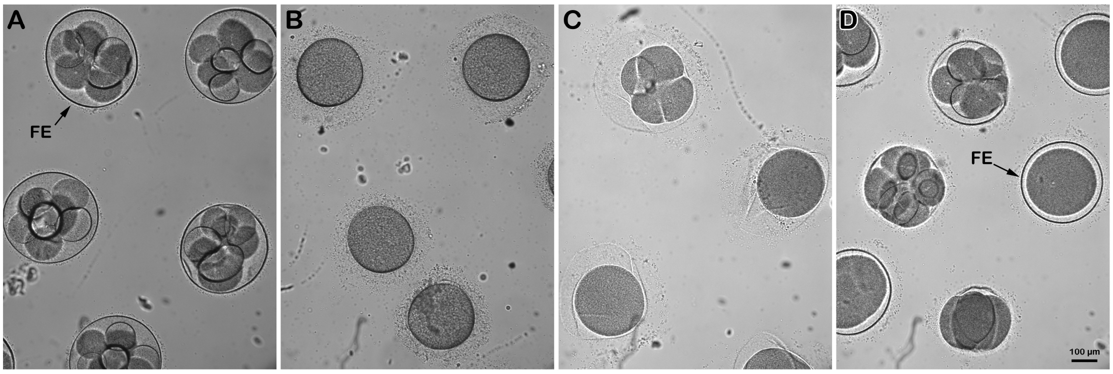

Figure S1: An early stage of *A. aranciacus* embryonic development. (**A**) Starfish embryos (4 h after insemination) derived from fertilization of immature oocytes stimulated with 1 MA for 70 min (mature egg), with a normal fertilization envelope (FE) elevation; (**B**) Immature oocytes treated with DTT for 70 min and inseminated failed to undergo cortical reaction and cleavage; (**C**) Cleavage impairment following fertilization of immature oocytes stimulated with 1-MA for 60 min to induce GVBD and 10 min with DTT; (**D**) Cleavage impairment in the eggs exposed to DTT (10 min) and washed in NSW before insemination. Elevation of the fertilization envelope (FE) in these zygotes appears normal despite the cleavage blockage.

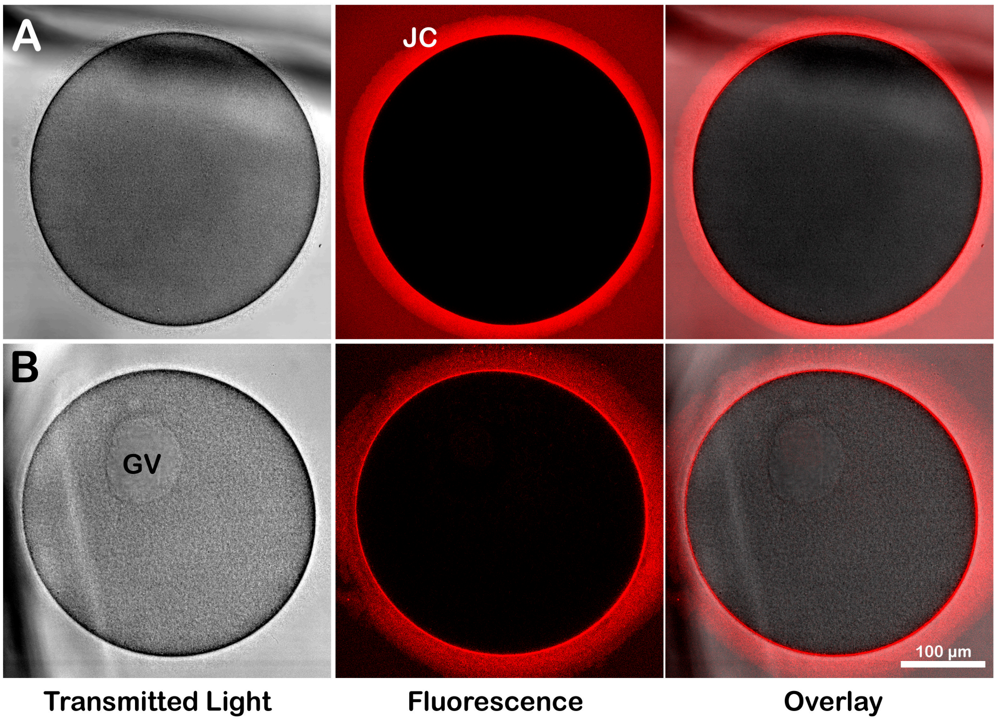

Figure S2: Live GV-stage oocytes were induced to undergo maturation by stimulation with 1-MA (**A**) or DTT (**B**) for 70 min. In the confocal transmitted light image, the oocyte incubated with 10 mM DTT still contains GV with delineated membranes. The confocal fluorescence and overlay images show the jelly coat (JC), which is disclosed by fluorescent polyamine marker BPA-C8-Cy5 (25 µM), appreciably reduced by the DTT treatment (5 min) with a less compact texture but still present.
